# Supplementary material for: Effects of paleogeographic changes and CO2 variability on northern mid-latitudinal temperature gradients in the Cretaceous
Source: Nat Commun. 2023 Aug 25;14:5193. doi: 10.1038/s41467-023-40905-7 (PMC10457304; doi:10.1038/s41467-023-40905-7)
Supplement: Supplementary file 1 — Supplementary Information [file 41467_2023_40905_MOESM1_ESM.pdf]

Supplementary information for

Effects of paleogeographic changes and CO_2 variability on northern mid-latitudinal temperature gradients in the Cretaceous

Kaushal Gianchandani[1*], Sagi Maor[1], Ori Adam[1], Alexander Farnsworth[2,3], Hezi Gildor[1], Daniel J. Lunt[2], Nathan Paldor[1]

[1]Fredy & Nadine Herrmann Institute of Earth Sciences, Hebrew University of Jerusalem, Edmond J. Safra Campus, Givat Ram, Jerusalem, 9190401, Israel

[2]School of Geographical Sciences, and Cabot Institute, University of Bristol, Bristol, BS8 1SS, UK

[3]State Key Laboratory of Tibetan Plateau Earth System, Environment and Resources (TPESER), Institute of Tibetan Plateau Research, Chinese Academy of Sciences, Beijing 100101, China.

*Corresponding author. E-mail: kaushal.g@mail.huji.ac.il

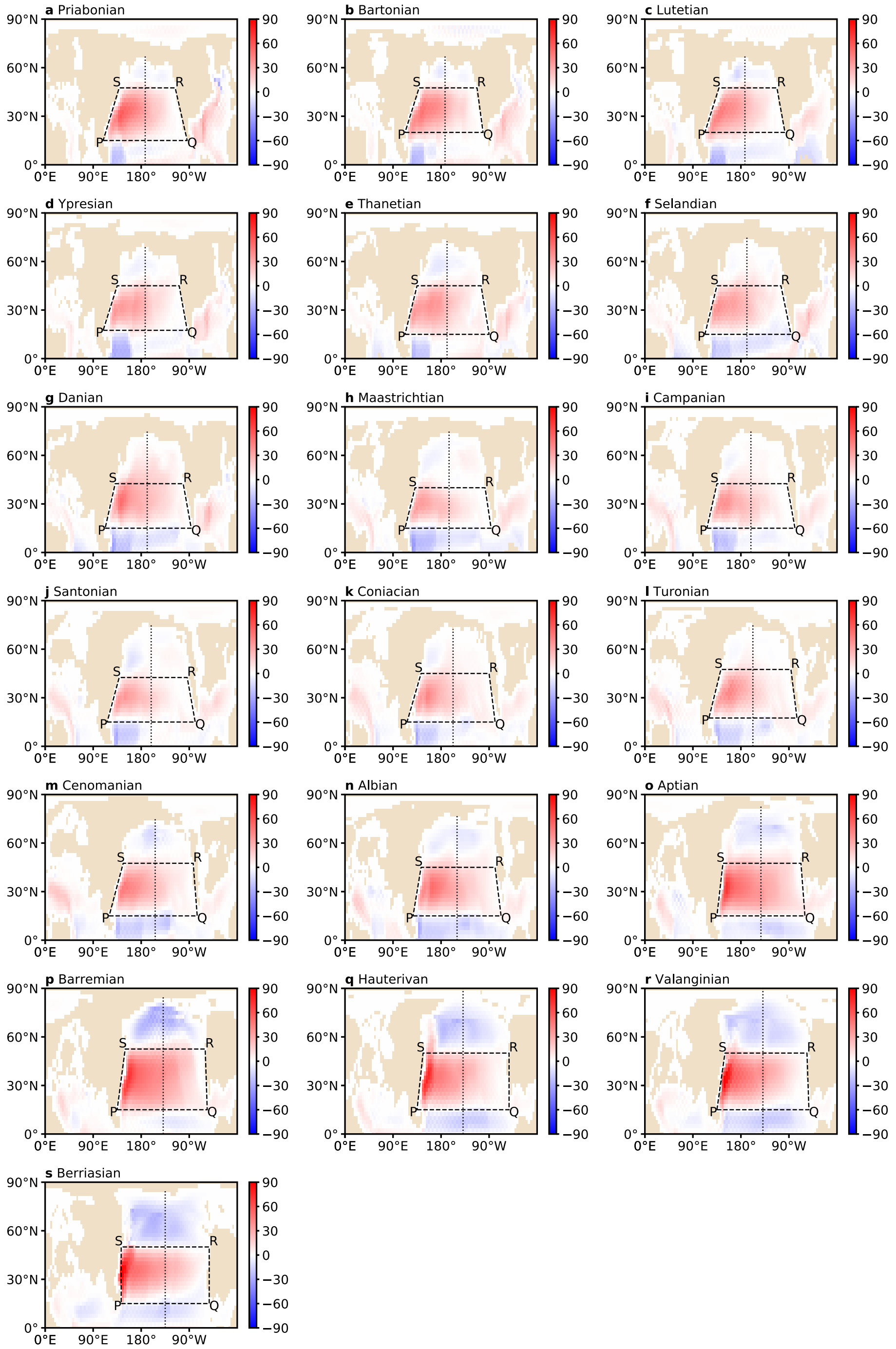

Supplementary Figure 1 | Stream function (Sv) and the mid-latitudinal basin in the paleo-Northern Pacific for each geological age during the Cretaceous and Paleogene. The dashed lines on each panel denote the trapezoidal basin (PQRS) constructed to estimate the typical meridional and zonal extents of the 'gyral basin' (L_x and L_y) and the dotted line denotes the longitude along which the typical pole-to-Equator extent of the ocean basin ($\widetilde{L_y}$) is estimated. **a** Priabonian, **b** Bartonian, **c** Lutetian, **d** Ypresian, **e** Thanetian, **f** Selandian, **g** Danian, **h** Maastrichtian, **i** Campanian, **j** Santonian, **k** Coniacian, **l** Turonian, **m** Cenomanian, **n** Albian, **o** Aptian, **p** Barremian, **q** Hauterivian, **r** Valanginian and **s** Berriasian.

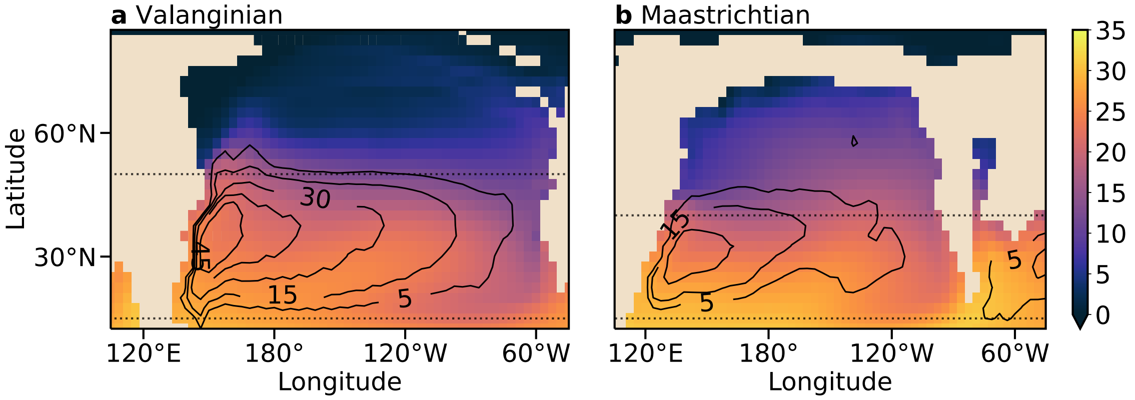

Supplementary Figure 2 | Surface temperature fields in the North Pacific Ocean during the Valanginian age (~135 Ma) and the Maastrichtian age (~68 Ma) for an atmospheric CO_2 concentration of 560 ppmv. **a** and **b** show sea surface temperature (°C, color) and streamlines (Sv, contour; 1 Sv = 10^6 m^3 s^{-1}) in the ocean during the two ages. The dotted lines mark the latitudes where the wind-stress curl is zero. For comparison with Fig. 1b-c in the main article which shows the same plots but for 1120 ppmv.

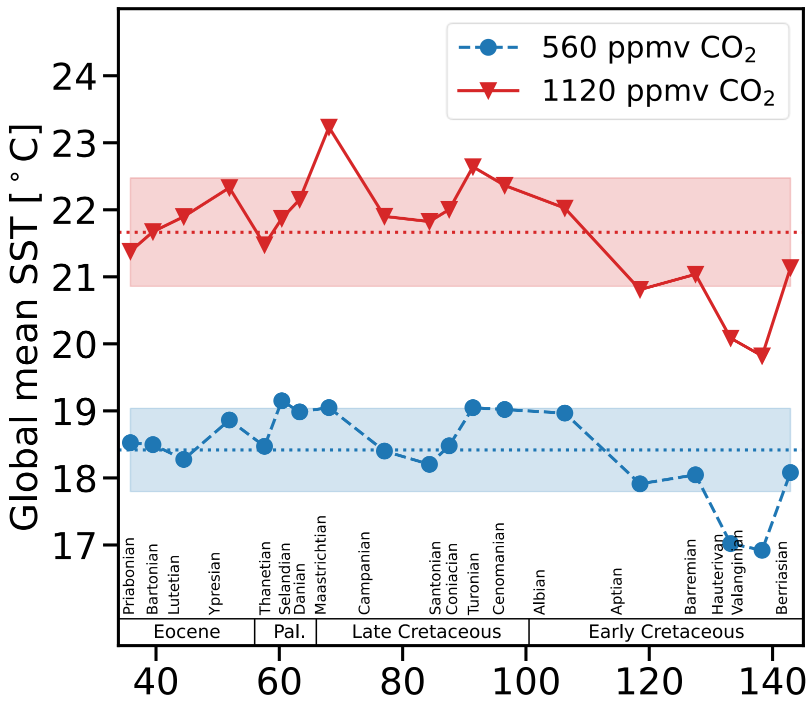

Supplementary Figure 3 | Global annual mean sea surface temperature (SST) for an atmospheric CO_2 concentration of 560 ppmv (dashed blue curve) and 1120 ppmv (solid red curve). The dotted lines indicate the average of global mean SST across each ensemble of simulations and the shaded regions denote the 1σ deviation from the respective averages.

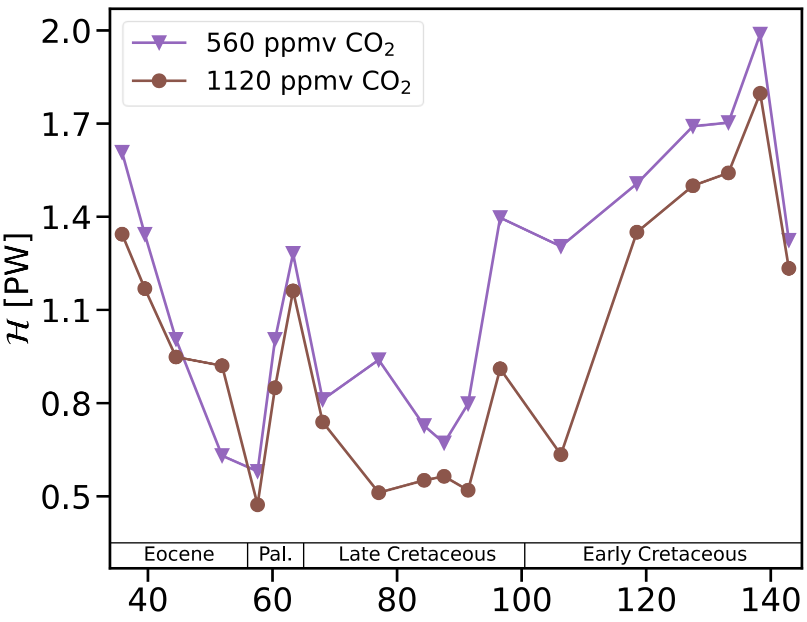

Supplementary Figure 4 | Surface heat transport associated with the ocean gyre in the mid-latitudinal northern paleo-Pacific basin.

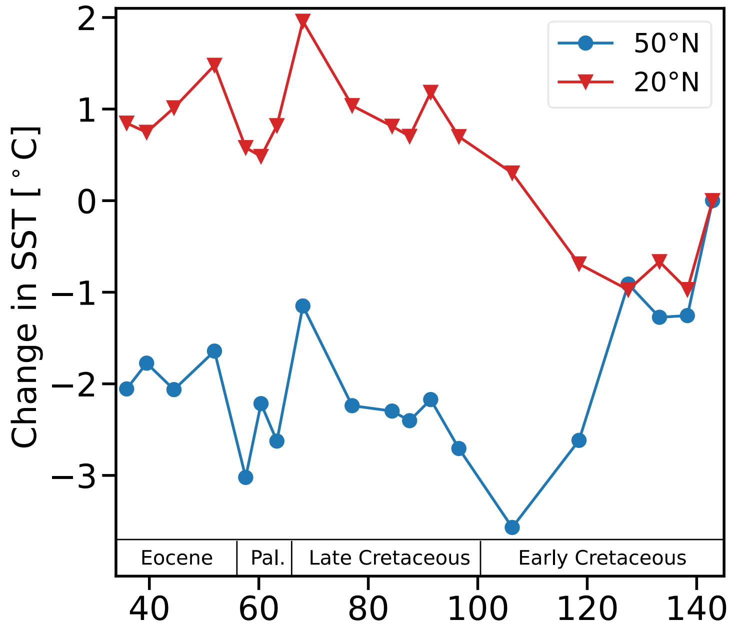

Supplementary Figure 5 | Zonally averaged sea surface temperature at 20°N and 50°N for different geological ages, expressed as an anomaly relative to the Berriasian age (~142 Ma).

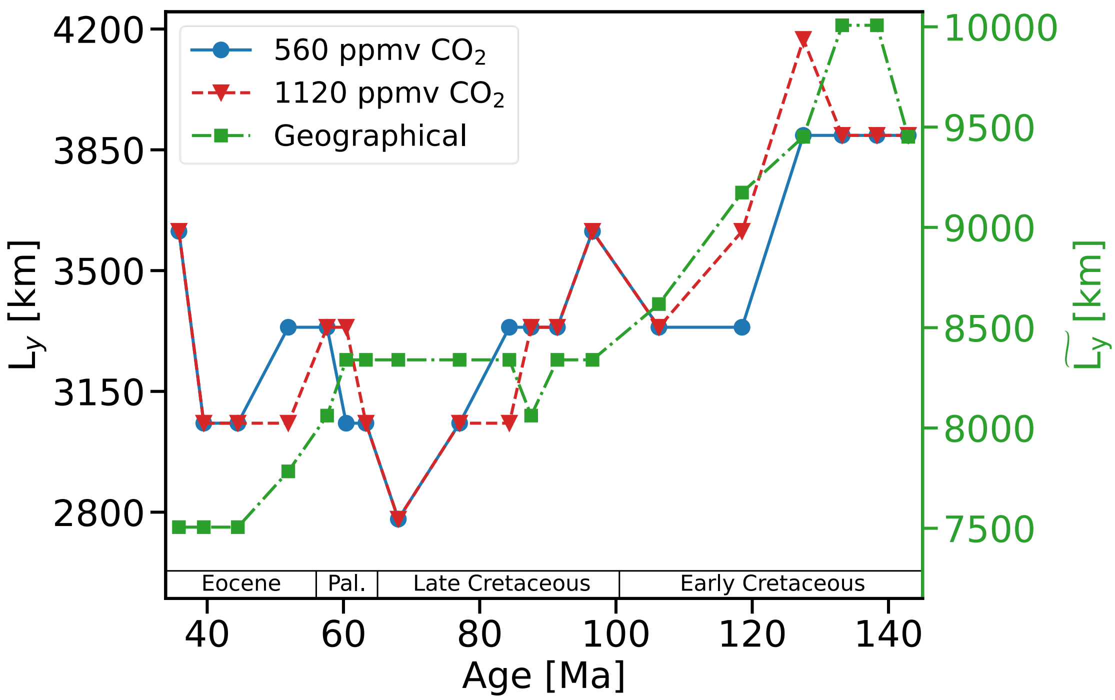

Supplementary Figure 6 | Changes in the meridional extent of gyral basin (L_y) for an atmospheric CO_2 concentration of 560 ppmv (solid blue curve) and 1120 ppmv (dashed red curve) and the pole-to-Equator extent of ocean basin ($\widetilde{L_y}$, dotted-dashed green curve) in the Northern paleo-Pacific from the Early Cretaceous to the Eocene. The dotted lines in each basin illustrated by Fig. S1 denotes the longitude along which $\widetilde{L_y}$ is calculated.

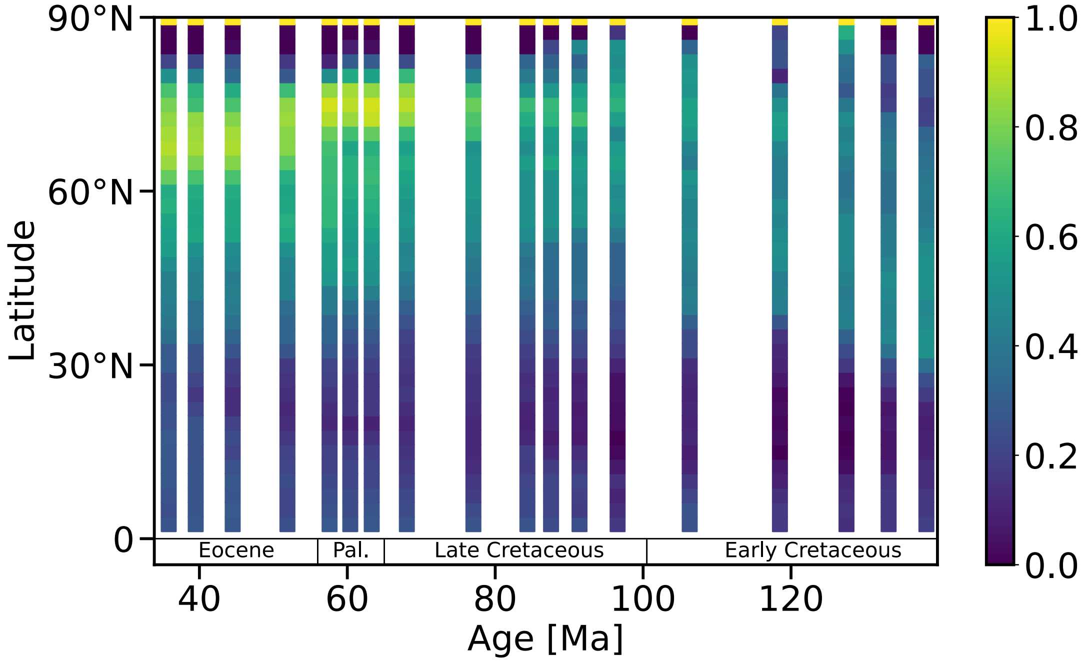

Supplementary Figure 7 | Fraction of land area in the northern-Paleo Pacific during the Cretaceous-Paleogene. The fraction of land area in the subpolar region increases from the mid-Cretaceous to the Eocene.
